# SMAD3 contributes to ascending aortic dilatation independent of transforming growth factor-beta in bicuspid and unicuspid aortic valve disease

**DOI:** 10.1038/s41598-022-19335-w

**Published:** 2022-09-14

**Authors:** Brittany Balint, Jan Federspiel, Catherine Kollmann, Paul Teping, Tanja Schwab, Hans-Joachim Schäfers

**Affiliations:** Department of Thoracic and Cardiovascular Surgery, Saarland University Medical Center, Kirrberger Str. 100, 66424 Homburg/Saar, Germany

**Keywords:** Aortic diseases, Aneurysm

## Abstract

We sought to determine whether there are differences in transforming growth factor-beta (TGFß) signaling in aneurysms associated with bicuspid (BAV) and unicuspid (UAV) aortic valves versus normal aortic valves. Ascending aortic aneurysms are frequently associated with BAV and UAV. The mechanisms are not yet clearly defined, but similarities to transforming growth factor-beta TGFß vasculopathies (i.e. Marfan, Loeys-Dietz syndromes) are reported. Non-dilated (ND) and aneurysmal (D) ascending aortic tissue was collected intra-operatively from individuals with a TAV (N = 10ND, 10D), BAV (N = 7ND, 8D) or UAV (N = 7ND, 8D). TGFß signaling and aortic remodeling were assessed through immuno-assays and histological analyses. TGFß1 was increased in BAV/UAV-ND aortas versus TAV (*P* = 0.02 and 0.04, respectively). Interestingly, TGFß1 increased with dilatation in TAV (*P* = 0.03) and decreased in BAV/UAV (*P* = 0.001). In TAV, SMAD2 and SMAD3 phosphorylation (pSMAD2, pSMAD3) increased with dilatation (all *P* = 0.04) and with TGFß1 concentration (*P* = 0.04 and 0.03). No relationship between TGFß1 and pSMAD2 or pSMAD3 was observed for BAV/UAV (all *P* > 0.05). pSMAD3 increased with dilatation in BAV/UAV aortas (*P* = 0.01), whereas no relationship with pSMAD2 was observed (*P* = 0.56). Elastin breaks increased with dilatation in all groups (all *P* < 0.05). In TAV, elastin degradation correlated with TGFß1, pSMAD2 and pSMAD3 (all *P* < 0.05), whereas in BAV and UAV aortas, elastin degradation correlated only with pSMAD3 (*P* = 0.0007). TGFß signaling through SMAD2/SMAD3 contributes to aortic remodeling in TAV, whereas TGFß-independent activation of SMAD3 may underlie aneurysm formation in BAV/UAV aortas. Therefore, SMAD3 should be further investigated as a therapeutic target against ascending aortic dilatation in general, and particularly in BAV/UAV patients.

## Introduction

Malformations of the aortic valve (AV) are associated with frequent occurrence of ascending aortic dilatation and dissection^1^. For individuals with a bicuspid aortic valve (BAV), the probability of aortic aneurysm is up to 80-fold higher compared to those with a normal (i.e. tricuspid) aortic valve (TAV)^2^. The risk of aortic complications may be even higher in individuals with a unicuspid aortic valve (UAV)^3^.

The etiology of ascending aortic aneurysms in patients with AV malformations remains incompletely understood. It is likely that its origin has a multifactorial nature, with turbulent blood flow and genetic factors playing a role^4–6^. This is in contrast to some heritable thoracic aortic diseases, including Marfan syndrome and Loeys-Dietz syndrome, in which monogenic mutations of genes involved in the transforming growth factor-beta (TGFß) signaling cascade have been identified^7^. Aberrant TGFß signaling is associated with vulnerable connective tissue in the aortic wall, which underlies aneurysm development. Although the mechanisms are unknown, evidence of increased TGFß signaling has also been observed in dilated BAV aortas^8,9^.

TGFß is a key regulator of vascular remodeling and smooth muscle cell (SMC) activity. Activation of canonical TGFß signaling occurs by ligand binding and signaling through serine/threonine receptor complexes, which then activate downstream signaling mediators, SMAD2 and SMAD3^10^. Phosphorylated SMAD2 and SMAD3 form complexes with co-SMADs and are translocated to the nucleus where they activate target SMAD-dependent genes^11^. Evidence from knockout studies revealed that SMAD2 and SMAD3 have both overlapping and distinct physiological roles^12^. SMAD2 and SMAD3 are often studied as mediators of TGFß signaling in aortopathy^13^, so it is uncertain whether they have differential mechanistic roles in ascending aortic aneurysm formation.

Regardless of etiology, most ascending aortic aneurysms appear to develop on the background of aortic wall remodeling. Commonly observed histopathological findings include disorganization of the media, elastin degradation, destructive changes to SMCs and mucoid extracellular matrix accumulation (MEMA)^14–16^. For BAV and UAV individuals, whether aortic remodeling is due to dysregulated TGFß signaling remains unknown.

In the current study, we evaluated canonical TGFß signaling in the ascending aorta from individuals with BAVs or UAVs, comparing them with TAV aortas. We also assessed for differences in signaling in order to define whether dysregulated TGFß signaling is associated with ascending aortic remodeling in BAV or UAV aortas.

## Materials and methods

The data that support the findings of this study are available from the corresponding author upon reasonable request. This study complies with the Declaration of Helsinski, and was carried out with approval from the regional ethics committee (Ständige Ethikkommission der Ärztekammer des Saarlandes, Proposal # 47/14). Informed consent was obtained from all patients included in this study.

### Procurement of ascending aortic tissue

Ascending aortic specimens were obtained intra-operatively from individuals with tricuspid (n = 20), bicuspid (n = 15) or unicuspid (n = 15) aortic valves at the time of thoracic aortic surgery. Individuals diagnosed with connective tissue disorders (e.g. Marfan syndrome, Loeys-Dietz syndrome) or with serological evidence of chronic viral diseases (e.g. HIV, Hepatitis B/C) were excluded. Samples with macroscopic evidence of inflammatory disease (e.g. atherosclerosis, aortitis) were also omitted. Aortic tissue was harvested from the anterior circumference of the ascending aorta, 5–10 mm above the sinotubular junction, and in the case of aneurysmal aortas, also from the mid-ascending aorta. Tissue samples extracted in the operating room were divided and either directly frozen in liquid nitrogen for later protein extraction or directly transferred to 4% phosphate-buffered formalin for immediate fixation.

Aortic valve morphology was determined pre-operatively by either trans-esophageal or trans-thoracic echocardiography, and was confirmed intra-operatively by the surgeon. Ascending aortic dimensions were measured by pre-operative computed tomography. Secondary measurements were taken intra-operatively by trans-esophageal echocardiography. If there was a discrepancy between measurements, an average of all available measurements was used in this study. With a threshold of 4 cm^17^, aneurysms were identified in 10/20 TAV patients (50%), 8/15 BAV patients (53.3%) and 8/15 UAV patients (53.3%).

### TGFß1 concentration by ELISA

Total protein was extracted from the ascending aorta using the protein quant sample lysis kit (Life Technologies, Nr. 4448536, Lot 00317896) according to the manufacturer’s instructions. A protease inhibitor (Protease Cocktail Set I, Calbiochem, Nr. 53913) and a phosphatase inhibitor (Phosphatase Cocktail Set II, Calbiochem, Nr. 524625) were used in the extraction. TGFß1 concentration was determined using a TGFß1 ELISA kit (Cloud-Cone Corporation, Nr. SEA124Hu, Lot L140701005) according to the manufacturer’s instructions. Protein samples were thawed on ice and diluted with assay buffer (1:1). Test samples were run in triplicate on a 96-well plate, alongside a standard curve of known TGFß1 concentrations. Test sample absorbance was measured at a wavelength of 450 nm and compared to that of the standard curve. TGFß1 concentration values are expressed as pg/100 µg protein.

### Histology

To assess for morphological evidence of remodeling, we studied the histology of the aortic wall from each patient. Formalin-fixed, paraffin-embedded aortic tissue samples were serially sectioned at 1 µm thickness. Aortic sections were labeled with the following stains according to the manufacturer’s instructions: Hematoxylin eosin, Elastica hematoxylin eosin, Toluidine-blue, Alcian blue, Masson–Goldner trichrome and Movat pentachrome (Verhoeff). Stained aortic tissue sections were visualized with an Olympus BX60 microscope. Images were taken with an attached Olympus DP37 camera. Analyses were carried out using cellSens Dimensions 1.15 (Build 14760; Copyright^©^ 2009–2016 OLYMPUS CORPORATION).

Elastin loss, medial fibrosis and mucoid extracellular matrix accumulation (MEMA) were graded based on the parameter definitions given by the consensus statement on non-inflammatory aortic disease^18^. Briefly, the severity of each endpoint was rated on a scale of 0–3, with 0 indicating no apparent pathology, and 3 indicating that > 10 lamellar units within the aortic wall were affected. A blinded primary examiner performed the analysis of elastin loss, medial fibrosis and MEMA. The results were confirmed by experienced anatomists and pathologists.

### Immunohistochemistry

Formalin-fixed, paraffin embedded aortic tissue sections were immunolabeled with rabbit polyclonal antibodies against TGFß1 (1:100, AB155264, Abcam) phosphorylated SMAD2 (1:30; AB53100, phospho S467, Abcam), phosphorylated SMAD3 (1:100, 44-246G, phospho S423/S425, Invitrogen), or fibrillin-1 (1:75; AB53076, Abcam). Bound primary antibodies were visualized using fluorescently conjugated secondary antibodies (goat anti-rabbit 647 (for TGFß1), goat anti-rabbit 488 (for pSMAD2 and pSMAD3) or goat anti-rabbit 647 (for fibrillin-1)). Sections were counterstained with DAPI. Images were captured using a laser scanning confocal microscope (Zeiss LSM, Plan Apochromat, 40×, 1.3 oil objective). For elastin breaks, elastin was measured by autofluorescence recorded by excitation at 488 nm in sections stained only with DAPI. To analyze each parameter, 10 regions of interest were captured for each patient. For pSMAD2 and pSMAD3, the number of positive cells were counted and normalized to the total number of cells in each image. A cell was considered positive if 5 or more fluorescent dots were observed in the cell nucleus. For TGFß1 and fibrillin-1, fluorescence intensity of the red channel was measured with ImageJ software (NIH). Elastin breaks were counted as the number of discrete regions of discontinuous elastin fibers per mm^2^ of aortic tissue.

### Statistics

Statistical analyses were carried out using Prism 7 (Graphpad Software). All data sets were tested for normality using the D’Agostino and Pearson omnibus test. Comparisons between control and test groups were carried out using the student’s t-test or the analysis of variance with Bonferroni’s post-hoc test (for normally distributed datasets) or with the Mann–Whitney U test or the Kruskal–Wallis and Dunn’s post-hoc test (for non-normal datasets). Relationships between parameters were assessed by employing linear regression analyses. Data are presented as mean ± standard error of the mean (SEM). Significance was set at *P* < 0.05.

## Results

### Clinical characteristics

Ascending aortic tissue was harvested intra-operatively from a total of 50 patients. Aortic tissue was characterized into six groups based on aortic valve morphology and maximal ascending aortic diameter: TAV-non-dilated (TAV-ND, n = 10), TAV-dilated (TAV-D, n = 10), BAV-non-dilated (BAV-ND, n = 7), BAV-dilated (BAV-D, n = 8), UAV-non-dilated (UAV-ND, n = 7) and UAV-dilated (UAV-D, n = 8). Clinical characteristics are presented in Tables 1 and 2. There was no significant difference in the proportion of patients presenting with primary aortic valve stenosis between groups (*P* = 0.67; Table 1). In the BAV group, the majority of patients presented with primary aortic valve insufficiency (*P* = 0.002), whereas combined aortic valve stenosis and insufficiency was predominantly observed in the TAV and UAV groups (*P* = 0.003; Table 1). The proportion of patients with hypertension was increased in the TAV versus BAV and UAV groups (*P* = 0.02; Table 1).

**Table 1:** Patient characteristics based on aortic valve morphology.

	Tricuspid	Bicuspid	Unicuspid	P value
N Number	20	15	15	
Age; mean ± standard deviation	62.6 ± 12.9	46.4 ± 15.5	35.8 ± 15.5	< 0.0001*
Sex; % male	80.0	86.7	73.3	0.41

	Aortic valve pathology; n (%)			
Stenosis	5 (25)	0 (0)	2 (13.3)	0.67
Insufficiency	5 (25)	14 (93.3)	3 (20)	0.002*
Combined	10 (50)	1 (6.7)	10 (66.7)	0.003*

	Co-morbidities; n (%)			
Hypertension	10 (50)	5 (33.3)	3 (20)	0.02*
Hyperlipidemia	3 (15)	2 (10)	3 (20)	0.62

	Medications; n (%)			
ß-Blocker	8 (40)	6 (40)	7 (46.7)	0.42
ACE-inhibitor	6 (30)	7 (46.7)	4 (26.7)	0.19
AT1-receptor antagonist	2 (10)	4 (26.7)	4 (26.7)	0.55
Diuretic	7 (35)	5 (33.3)	2 (10)	0.04*
Calcium channel blocker	3 (15)	1 (6.6)	0 (0)	0.08
Statin	7 (35)	4 (26.7)	3 (20)	0.31
Aldosterone antagonist	2 (10)	0 (0)	0 (0)	0.22

*Statistical significance.

**Table 2:** Patient characteristics based on aortic diameter.

	Tricuspid		Bicuspid		Unicuspid	
	Normal	Dilated	Normal	Dilated	Normal	Dilated
N number	10	10	7	8	7	8
Age; y ± range	70 ± 11.7	55.1 ± 9.7	39.6 ± 16.6	54.3 ± 10.2	28.6 ± 16.8	44.2 ± 8.8
Sex; % male	80	70	100	75	71.4	75
**Maximal ascending aortic diameter**						
Mean ± SD	3.5 ± 0.1	6.1 ± 1.6	3.5 ± 0.3	4.8 ± 0.2	2.9 ± 0.7	4.9 ± 0.2
Range	3.3 – 3.7	5.0 – 8.1	3.0 – 3.9	4.5 – 5.0	2.0 – 3.7	4.6 – 5.3

### TGFß ligand concentration

**Figure 1:**
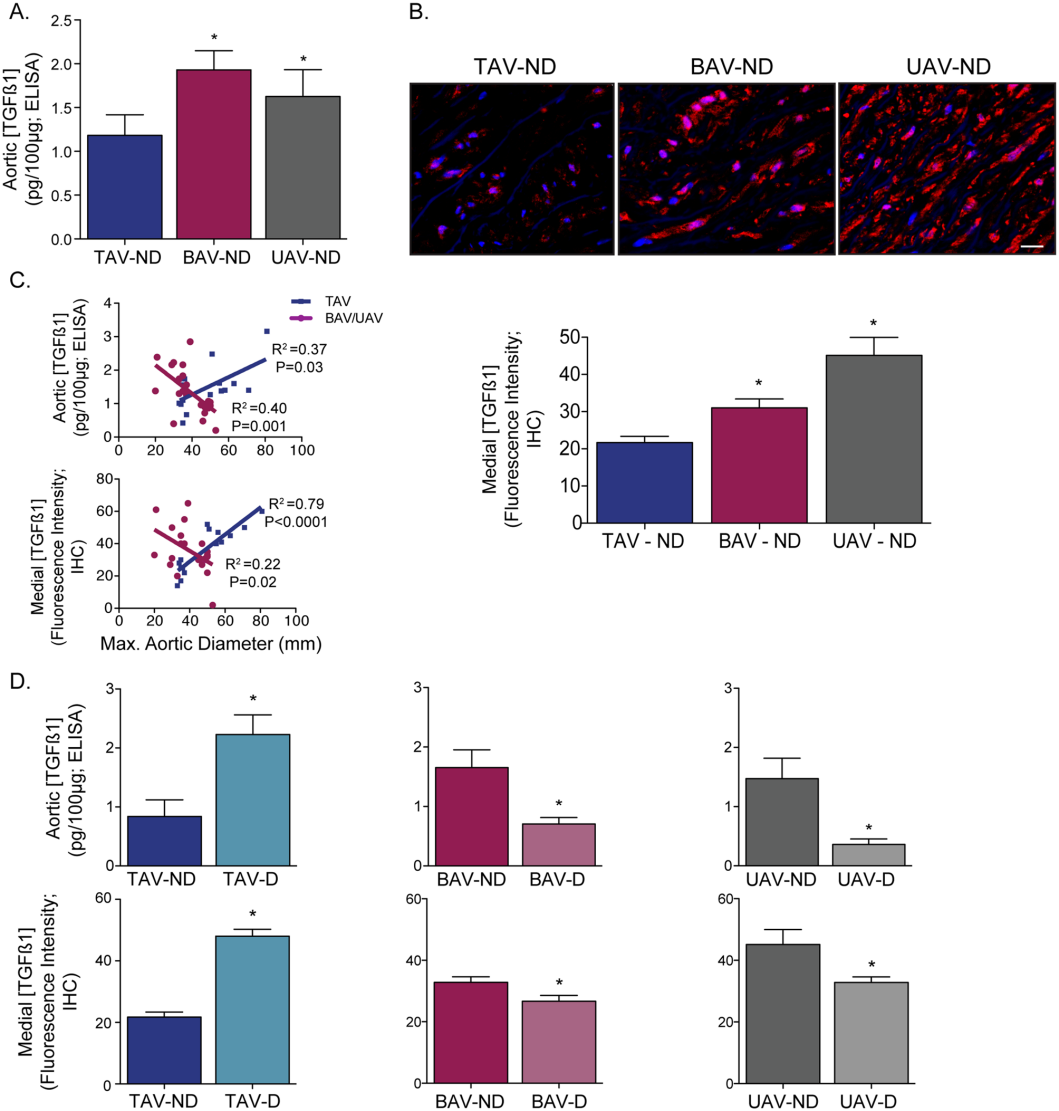
Transforming growth factor-beta (TGFß) ligand concentration correlates to ascending aortic diameter in tricuspid (TAV), but not bicuspid (BAV) or unicuspid (UAV) aortic valve patients. (**A**) Graph depicting the TGFß1 ligand concentration in non-dilated (ND) aortas from individuals with a TAV, BAV or UAV. (**B**) Fluorescent micrographs (top; blue = DAPI, red = TGFß1) and corresponding graph (bottom) depicting the TGFß1 concentration in the aortic media from individuals with ND aortas and either a TAV, BAV or UAV. (**C**) Graphs depicting the relationship between TGFß ligand concentration (whole aorta: top, media: bottom) and maximal ascending aortic diameter in TAV (blue) and BAV/UAV (pink) aortas. (**D**) Graphs depicting the TGFß1 ligand concentration (whole aorta: top, media: bottom) in ND and D aortas from individuals with a TAV, BAV or UAV. *P < 0.05. IHC = immunohistochemistry. Scalebar = 20 µm.

When comparing non-dilated aortas between AV groups, we found that TGFß1 ligand concentration from whole aortic samples was higher in individuals with malformed aortic valves (BAV: 1.93 ± 0.22, UAV: 1.63 ± 0.31) compared to those with TAVs (1.18 ± 0.23; *P* = 0.02 and 0.04, respectively; Fig. 1A). Immunostaining of TGFß1 confirmed a similar pattern, with higher TGFß1 concentrations in BAV and UAV aortas (*P* = 0.02 and *P* = 0.001 vs. TAV, respectively), particularly localized to the medial layer (Fig. 1B). There was no significant difference in TGFß ligand concentration in non-dilated aortas between BAV and UAV (*P* = 0.44; Fig. 1A,B). In individuals with a normal AV (TAV), TGFß ligand concentration increased with ascending aortic diameter in the whole aorta (R^2^ = 0.37; *P* = 0.03) and in the medial layer (R^2^ = 0.79; *P* < 0.0001; Fig. 1C) and the TGFß ligand concentration was higher in aneurysmal aortas (*P* = 0.01; Fig. 1D). Conversely, TGFß concentration actually decreased with ascending aortic diameter in BAV and UAV whole aortas (R^2^ = 0.4, *P* = 0.001) and in the medial layer (R^2^ = 0.22, *P* = 0.02; Fig. 1C). For BAV and UAV, the TGFß ligand concentration was decreased in aneurysmal aortas compared to non-dilated aortas (all *P* = 0.01; Fig. 1D).

### Phosphorylated SMAD2 and SMAD3

**Figure 2:**
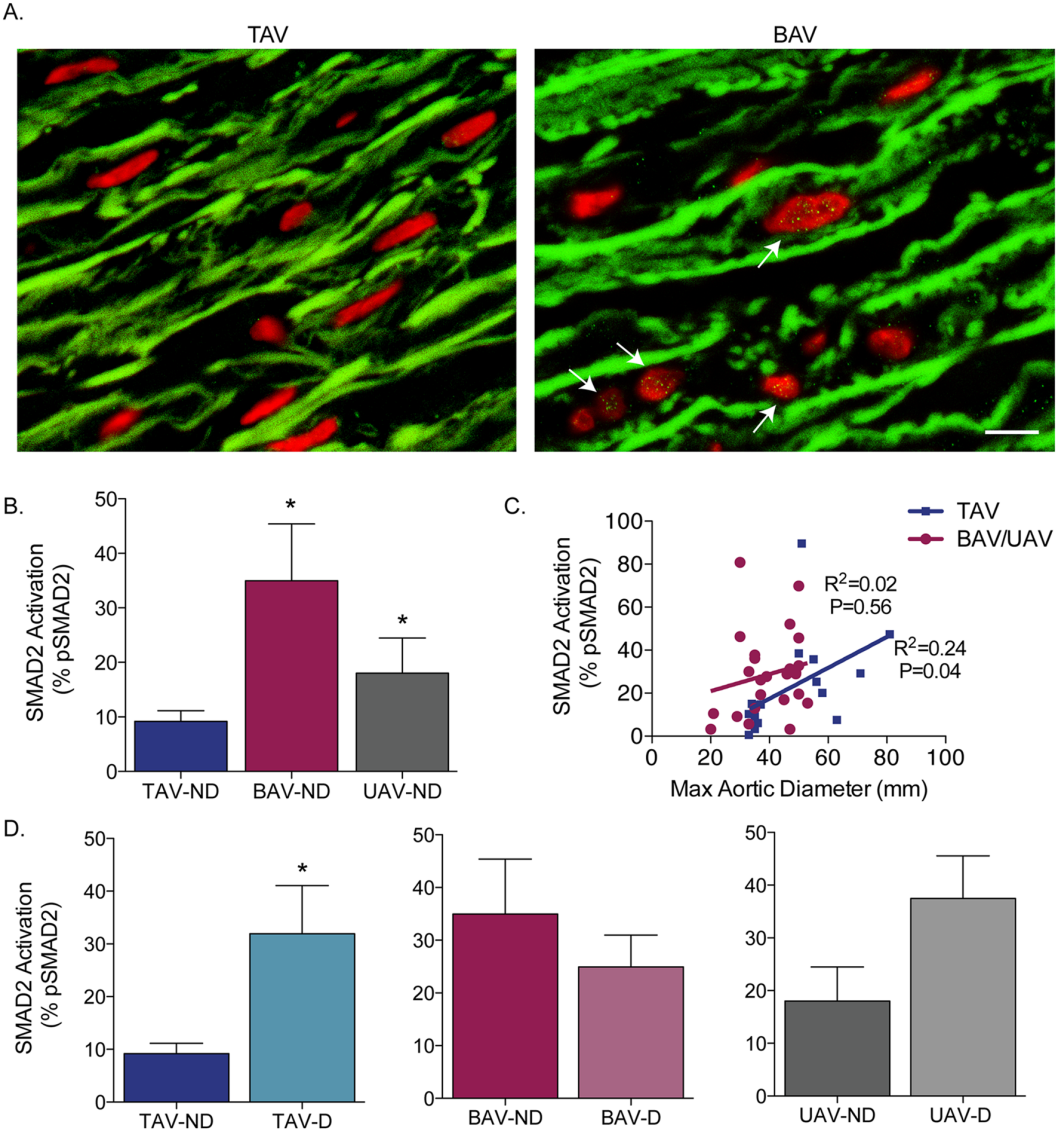
SMAD2 phosphorylation is increased in non-dilated bicuspid (BAV) and unicuspid (UAV) aortic valve aortas, and increases with dilatation in tricuspid aortic valve (TAV) aortas. (**A**) Fluorescent micrographs of ascending aortic cross-sections immunolabeled for phosphorylated SMAD2 (green points inside nuclei, indicated by white arrows) and counter-stained with DAPI (red). (**B**) Graph depicting the percentage of phosphorylated SMAD2 (pSMAD2)-positive smooth muscle cells in non-dilated (ND) aortas from individuals with a TAV, BAV or UAV. (**C**) Graph depicting the relationship between pSMAD2 and maximal ascending aortic diameter in TAV (blue) and BAV/UAV (pink) aortas. (**D**) Graphs depicting the percentage of pSMAD2-positive SMCs in ND and D aortas from individuals with a TAV, BAV or UAV. *P < 0.05. Scalebar = 10 µm.

As aortic TGFß ligand concentration differed between AV groups, we next assessed for disparities in downstream signaling. We analyzed the levels of both phosphorylated SMAD2 (pSMAD2) and phosphorylated SMAD3 (pSMAD3) in medial SMCs as a measure of downstream canonical TGFß signaling. In non-dilated aortas, the level of pSMAD2 was significantly increased in BAV (34.94 ± 10.40%) and UAV (18.04 ± 6.5%) aortas compared to TAV aortas (9.19 ± 1.95%, *P* = 0.016 and 0.04, respectively; Fig. 2A,B). There was a trend towards increased pSMAD2 in non-dilated BAV aortas versus UAV (*P* = 0.09; Fig. 2B). In TAV aortas, pSMAD2 increased with the maximal ascending aortic diameter (R^2^ = 0.24 *P* = 0.04; Fig. 2C), and the percentage of pSMAD2 SMCs was higher in aneurysmal aortas (*P* = 0.02, Fig. 2D). In BAV and UAV aortas, however, there was no significant correlation between pSMAD2 and ascending aortic diameter (R^2^ = 0.02, *P* = 0.56). For BAV, there was no change in pSMAD2 between dilated and non-dilated aortas (*P* = 0.42; Fig. 2D). In UAV aortas, however, there was a trend towards increased pSMAD2 in aneurysmal aortas (*P* = 0.08; Fig. 2D).

**Figure 3:**
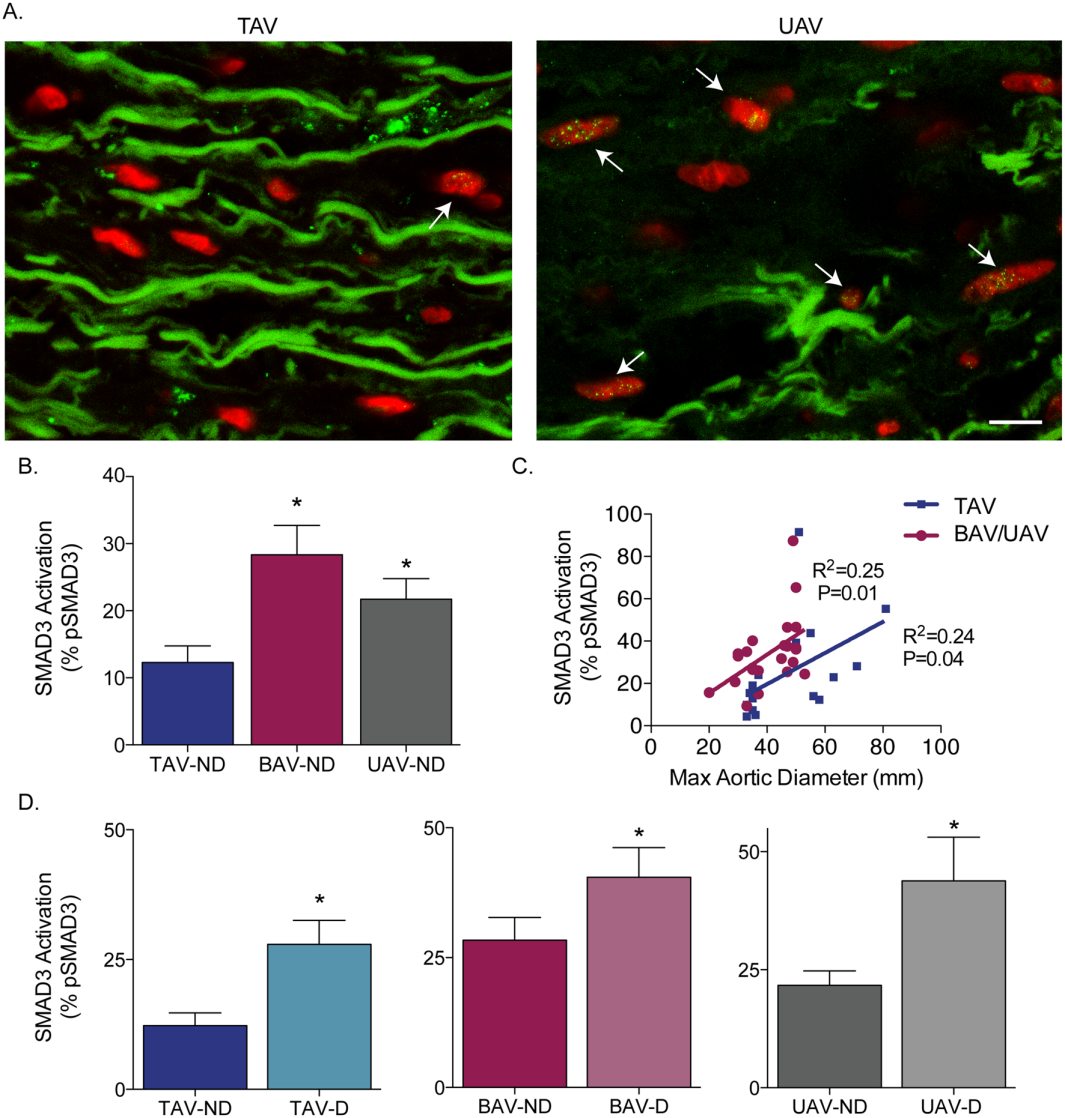
SMAD3 phosphorylation is increased in non-dilated bicuspid (BAV) and unicuspid (UAV) aortic valve aortas, and increases with dilatation in tricuspid aortic valve (TAV) and BAV/UAV aortas. (**A**) Fluorescent micrographs of ascending aortic cross-sections immunolabeled for phosphorylated SMAD3 (green points inside nuclei, indicated by white arrows) and counter-stained with DAPI (red). (**B**) Graph depicting the percentage of phosphorylated SMAD3 (pSMAD3)-positive smooth muscle cells in non-dilated (ND) aortas from individuals with a TAV, BAV or UAV. (**C**) Graph depicting the relationship between pSMAD3 and maximal ascending aortic diameter in TAV (blue) and BAV/UAV (pink) aortas. (**D**) Graphs depicting the percentage of pSMAD3-positive SMCs in ND and D aortas from individuals with a TAV, BAV or UAV. *P < 0.05. Scalebar = 10 µm.

In analyzing SMAD3 activation in normal-sized ascending aortas, we found a significant increase in pSMAD3 in BAV (28.34 ± 4.38%) and UAV (21.71 ± 3.08%) compared to TAV (12.28 ± 2.46%, *P* = 0.005 and 0.03, respectively; Fig. 3A,B). pSMAD3 did not differ between BAV and UAV ND aortas (*P* = 0.24; Fig. 3B). In TAV aortas, pSMAD3 positively correlated to maximal ascending aortic diameter (R^2^ = 0.24, *P* = 0.04; Fig. 3C), and aneurysmal TAV aortas had increased pSMAD3 compared to normal-sized aortas (*P* = 0.008; Fig. 3D). In contrast to SMAD2, dilatation seemed to have a significant impact on the level of activated SMAD3 in individuals with BAV or UAV aortas as the level of pSMAD3 positively correlated to the maximal ascending aortic diameter (R^2^ = 0.25, *P* = 0.01; Fig. 3C). Furthermore, aneurysmal aortas had higher pSMAD3 compared to non-dilated aortas in BAV (*P* = 0.03) and UAV (*P* = 0.04; Fig. 3D).

A subanalysis of the TAV dilated group revealed that 50% (5/10) of the patients had a tubular aortic aneurysm, while the remaining 50% (5/10) had an aortic root aneurysm, similar to that seen with connective tissue disorders^19^. Within the TAV dilated individuals there was a trend towards decreased age and increased SMAD3 phosphorylation in those with root aneurysms compared to those with tubular aneurysms (*P* = 0.10 and 0.08, respectively; Supplemental Fig. 1).

### pSMAD2 and pSMAD3 mediate TGFß signaling in TAV, but not in BAV or UAV aortas

**Figure 4:**
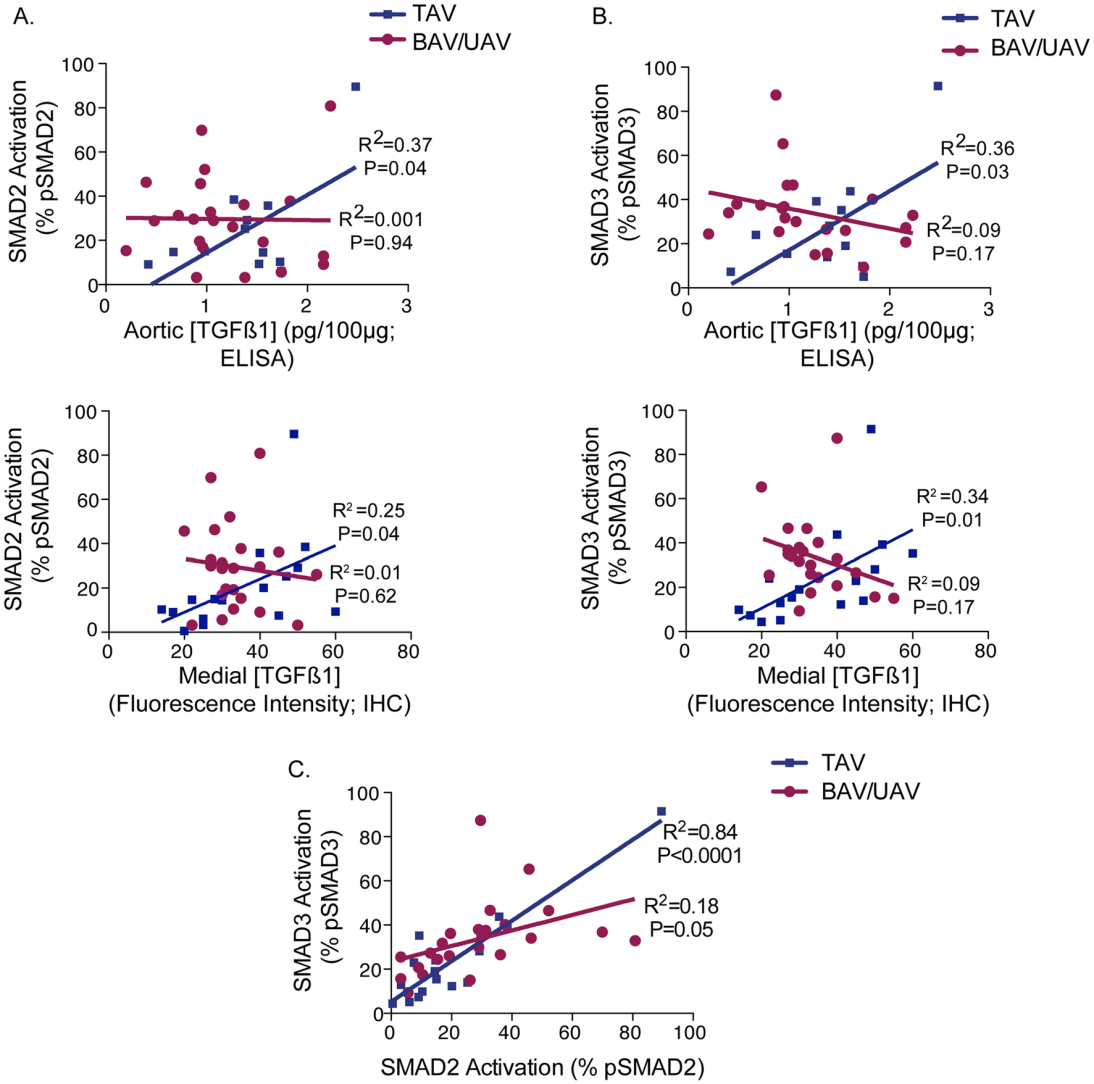
Phosphorylation of SMAD2 and SMAD3 is dependent upon TGFß1 ligand concentration in TAV, but not BAV/UAV aortas. (**A**) Graphs depicting the relationship between TGFß1 ligand concentration (whole aorta: top, medial layer: bottom) and phosphorylated SMAD2 in TAV (blue) and BAV/UAV (pink) aortas. (**B**) Graphs depicting the relationship between TGFß1 ligand concentration (whole aorta: top, medial layer: bottom) and phosphorylated SMAD3 in TAV (blue) and BAV/UAV (pink) aortas. (**C**) Graph depicting the relationship between phosphorylated SMAD2 and phosphorylated SMAD3 in TAV (blue) and BAV/UAV (pink) aortas.

We next assessed for relationships between TGFß ligand concentration and the downstream activation of SMAD2/3 in the ascending aorta. In individuals with a TAV, there was a positive correlation between TGFß1 concentration and the level of pSMAD2 (whole aorta: R^2^ = 0.37, *P* = 0.04, medial layer: R^2^ = 0.25, *P* = 0.04; Fig. 4A) and pSMAD3 (whole aorta: R^2^ = 0.36, *P* = 0.03, medial layer: R^2^ = 0.34, *P* = 0.01; Fig. 4B). Interestingly, however, no significant relationships were detected between TGFß1 ligand concentration and either pSMAD2 (whole aorta: R^2^ = 0.001, *P* = 0.94, medial layer: R^2^ = 0.01, *P* = 0.62) or pSMAD3 (whole aorta: R^2^ = 0.09, *P* = 0.17, medial layer: R^2^ = 0.09, *P* = 0.17) in BAV or UAV individuals (Fig. 4A,B). Assessing for relationships between pSMAD2 and pSMAD3 revealed a significant correlation in TAV aortas (R^2^ = 0.84, *P* < 0.0001), whereas a less pronounced relationship in BAV and UAV aortas was observed (R^2^ = 0.18, *P* = 0.05; Fig. 4C).

### Elastin degradation—independent of TGFB1 ligand concentration in BAV/UAV

**Figure 5:**
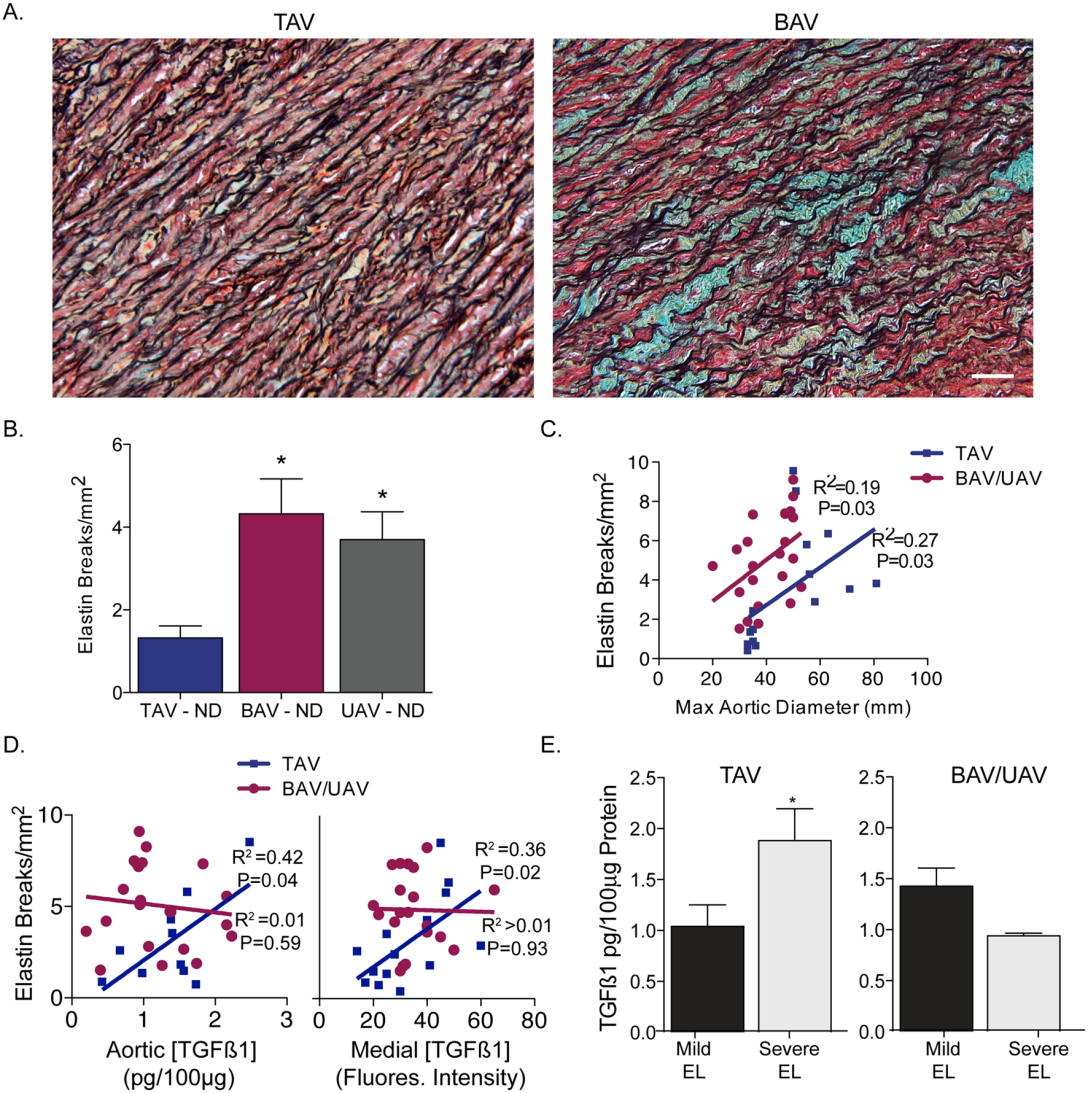
Elastin degradation is dependent upon transforming growth factor-beta ligand in tricuspid (TAV), but not bicuspid (BAV) or unicuspid (UAV) aortic aneurysms. (**A**) Photomicrographs of ascending aortic cross-sections labeled with Movat’s pentachrome, depicting elastin (black filaments) breaks and degradation in BAV aortas. Blue stain represents mucoid extracellular matrix deposition. (**B**) Graph depicting elastin breaks in non-dilated (ND) aortas from individuals with a TAV, BAV or UAV. (**C**) Graph depicting the relationship between elastin breaks and maximal ascending aortic diameter in TAV (blue) and BAV/UAV (pink) aortas. (**D**) Graphs depicting the relationships between TGFß1 ligand concentration (whole aorta: left, medial layer: right) and elastin breaks in TAV (blue) and BAV/UAV (pink) aortas. (**E**) Graphs depicting the TGFß1 ligand concentration in ascending aortas with mild elastin loss (EL) and severe EL for TAV (left) and BAV/UAV (right) aortas. *P < 0.05. Scalebar = 50 µm. Fluores. = fluorescence.

We next assessed for elastin degradation in normal and dilated ascending aortas. In non-dilated aortas, the number of elastin breaks/mm^2^ was increased in BAV (4.32 ± 0.85) and UAV (3.70 ± 0.68) compared to TAV aortas (1.3 ± 0.23; *P* = 0.003 and 0.004, respectively; Fig. 5A,B). As expected, the number of elastin breaks increased with dilatation in TAV aortas (R^2^ = 0.27, *P* = 0.03), and also in BAV and UAV aortas (R^2^ = 0.19, *P* = 0.03; Fig. 5C).

We next analyzed whether TGFß ligand concentration in the ascending aorta is associated with elastin degradation. In TAV aortas, the level of TGFß1 significantly correlated to the number of elastin breaks/mm^2^ (whole aorta: R^2^ = 0.42, *P* = 0.04, medial layer: R^2^ = 0.36, *P* = 0.02; Fig. 5D). In contrast, there was no detectable relationship between TGFß1 ligand concentration and the number of elastin breaks in BAV or UAV aortas (whole aorta: R^2^ = 0.01, *P* = 0.59, medial layer: R^2^ = 0.0004, *P* = 0.93; Fig. 5D). In addition to elastin breaks, elastin loss was graded in each sample. For TAV, TGFß1 concentration was higher in aortas with severe elastin loss compared to those with mild or no elastin loss (*P* = 0.04; Fig. 5E). On the other hand, no difference in TGFß1 ligand concentration was observed between aortas with mild or severe elastin loss in BAV or UAV aortas (*P* = 0.13; Fig. 5E).

### Fibrillin-1 concentration

Given its role in Marfan syndrome and its importance in elastin integrity, we assessed for levels of fibrillin-1 in the aorta. In normal-sized ascending aortas, the level of fibrillin-1 was significantly decreased in BAV and UAV aortas compared to TAV aortas (all *P* < 0.0001; Supplemental Fig. 2). The level of fibrillin-1 decreased with ascending aortic dilatation in TAV (R^2^ = 0.63, *P* = 0.0003), whereas no relationship was observed between fibrillin-1 and diameter for BAV and UAV aortas (R^2^ = 0.04, *P* = 0.34; Supplemental Fig. 2). Interestingly, no relationships were observed in either TAV or BAV/UAV aortas between fibrillin-1 and elastin breaks (TAV: R^2^ = 0.18, *P* = 0.10; BAV/UAV: R^2^ = 0.08, *P* = 0.17), TGFß1 concentration (TAV: R^2^ = 0.10, *P* = 0.32; BAV/UAV: R^2^ = 0.02, *P* = 0.53), pSMAD2 (TAV: R^2^ = 0.003, *P* = 0.85; BAV/UAV: R^2^ = 0.001, *P* = 0.86) or pSMAD3 (TAV: R^2^ = 0.01, *P* = 0.78; BAV/UAV: R^2^ = 0.01, *P* = 0.59; Supplemental Fig. 2).

### pSMAD3 versus pSMAD2 and elastin degradation

**Figure 6:**
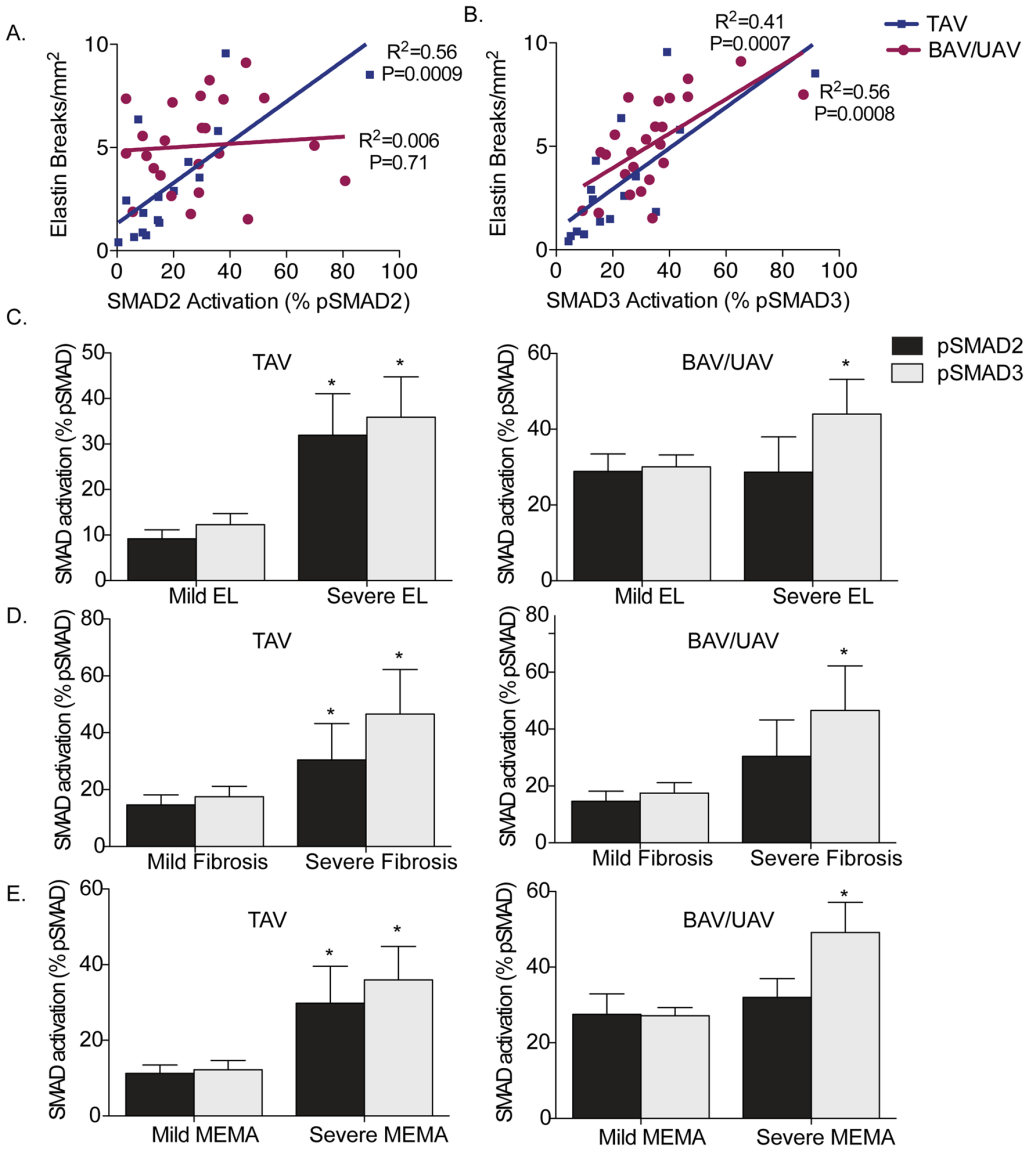
Aortic remodeling is dependent upon phosphorylation of SMAD3, but not SMAD2 in bicuspid (BAV) and unicuspid (UAV) aortic valve-associated ascending aortic aneurysms. (**A**) Graph depicting the relationship between elastin breaks and phosphorylated SMAD2 (pSMAD2) in TAV (blue) and BAV/UAV (pink) aortas. (**B**) Graph depicting the relationship between elastin breaks and phosphorylated SMAD3 (pSMAD3) in TAV (blue) and BAV/UAV (pink) aortas. (**C**–**E**) Graphs depicting the level of pSMAD2 (dark grey) and pSMAD3 (light grey) in ascending aortas with mild or severe elastin loss (EL; **C**), fibrosis (**D**) and mucoid extracellular matrix accumulation (MEMA; **E**) in TAV (left) and BAV/UAV (right) aortas. *P < 0.05.

To determine whether TGFß signaling through SMAD activation contributes to elastin degradation in the ascending aorta, we assessed for relationships between pSMAD2, pSMAD3 and features of elastin degradation. In individuals with a TAV, the number of elastin breaks/mm^2^ strongly correlated to both pSMAD2 (R^2^ = 0.56, *P* = 0.0009) and pSMAD3 (R^2^ = 0.56, *P* = 0.0008; Fig. 6A,B). In BAV and UAV aortas, however, there was no significant correlation between pSMAD2 and elastin breaks (R^2^ = 0.006, *P* = 0.71; Fig. 6A). On the other hand, there was a notable correlation between pSMAD3 and elastin breaks (R^2^ = 0.41, *P* = 0.0007; Fig. 6B). In TAV aortas, both pSMAD2 and pSMAD3 were higher in ascending aortas with severe elastin loss compared to mild or no elastin loss (*P* = 0.01 and 0.004, respectively; Fig. 6C). For BAV and UAV aortas, interestingly, pSMAD3 levels were higher in ascending aortas with severe elastin loss (*P* = 0.02), whereas there was no difference in pSMAD2 levels between aortas with mild or severe elastin loss (*P* = 0.27; Fig. 6C). These findings indicate the possibility of pSMAD3-specific mediation of elastin degradation in the aorta of individuals with malformed AVs.

### pSMAD3 versus pSMAD2 and medial degeneration

We next sought to determine whether activated SMAD3 could also contribute to extracellular matrix remodeling in the ascending aorta. There was no difference in the level of medial fibrosis in non-dilated aortas between groups (*P* = 0.87). For TAV and BAV, fibrosis was increased in dilated versus non-dilated aortas (*P* = 0.03 and *P* = 0.04, respectively). In UAV aortas, however, the level of fibrosis did not change with dilatation (*P* = 0.66). In ascending aortas with relevant medial fibrosis regardless of valve group, no differences were observed in TGFß1 concentration (*P* = 0.97). In TAV aortas, pSMAD2 (*P* = 0.02) and pSMAD3 (*P* = 0.01) levels were increased in aortas with relevant medial fibrosis (Fig. 6D). In BAV and UAV aortas on the other hand, pSMAD3 was increased in ascending aortas with relevant medial fibrosis (*P* = 0.04), whereas no change in pSMAD2 was observed (P = 0.21, Fig. 6D).

Similar to fibrosis, there was no difference in the level of MEMA in non-dilated aortas between groups (*P* = 0.49). In analyzing aneurysmal aortas, however, we found that MEMA increased with dilatation in TAV (*P* = 0.0005), BAV (*P* = 0.03) and UAV aortas (*P* = 0.005). When comparing aortas with and without relevant MEMA, no differences were observed in TGFß1 (*P* = 0.22). In TAV aortas with relevant MEMA, pSMAD2 and pSMAD3 were increased (*P* = 0.004 and 0.01, respectively; Fig. 6E). Interestingly, however, in BAV/UAV ascending aortas with relevant MEMA, pSMAD2 levels were unchanged (*P* = 0.21), while pSMAD3 levels were significantly increased in comparison to those without relevant MEMA (*P* = 0.0008; Fig. 6E). These findings support the hypothesis that the TGFß signaling cascade is involved in ascending aortic remodeling. Specific to ascending aortic aneurysms associated with AV malformations, however, SMAD3 may be a promising target.

## Discussion

Malformations of the aortic valve (i.e. bicuspid or unicuspid morphology) are associated with dilatation of the ascending aorta, although the mechanisms are not yet clearly defined. As TGFß signaling has been implicated in aortic dilatation related to heritable connective tissue disorders (i.e. Marfan, Loeys-Dietz), we assessed whether it is also associated with BAV or UAV aortic aneurysms. In the current study, we found evidence to suggest that there are distinct differences in the TGFß activation process in BAV and UAV aortas compared to TAV. By evaluating SMAD2 and SMAD3 activation, we found evidence for TGFß-independent activation of SMAD3, which correlates with aortic wall remodeling in BAV and UAV aortas.

Similar to findings in Marfan^20,21^ and Loeys-Dietz^22^ syndromes, we found that TGFß ligand concentration increased with dilatation in TAV aortas. This was accompanied by a decrease in fibrillin-1 with dilatation in TAV aortas, which is in line with findings in Marfan syndrome^23^. Patients with known connective tissue disorders were excluded from our study, so it is particularly noteworthy that aneurysm formation in the TAV group shared similarities with Marfan and Loeys-Dietz patients, albeit less pronounced. A subanalysis revealed that half of the TAV dilated patients in this study presented with a root aneurysm, similar to what is seen with connective tissue disorders. Despite a trend towards being younger, TAV patients with a dilated aortic root showed a pattern of more pronounced pathology compared to those with a tubular aortic aneurysm. These findings suggest that patients with a TAV and aortic root dilatation have similarities to those with connective tissue disorders, and should possibly be treated similarly. As our population in the sub-analysis was limited (n = 10), further work should be done to define the pathological differences between TAV individuals with dilated aortic roots compared to those with tubular aneurysms.

On the other hand, although TGFß was elevated in non-dilated BAV/UAV aortas versus TAV, we found that TGFß concentration actually decreased with dilatation in BAV and UAV aortas. This is consistent with the previous finding of elevated TGFß1 mRNA in non-dilated BAV aortas compared to TAV, which was decreased in dilated aortas^24^. Furthermore, we found that fibrillin-1 was decreased in non-dilated BAV and UAV aortas compared to TAV, which may contribute to the elevated TGFß1 ligand concentration at baseline. These findings suggest that there are distinct differences in TGFß signaling in the ascending aorta of individuals with a BAV or UAV compared to TAV, and that these signaling differences exist prior to aneurysm formation.

The relationship between TGFß ligand concentration and SMAD activation showed distinctively different patterns between TAV and BAV/UAV aortas. In the current study, we found that the level of pSMAD2 significantly increased with dilatation in TAV aortas. Furthermore, pSMAD2 positively correlated with medial TGFß ligand concentration, suggesting that SMAD2-dependent TGFß signaling plays a role in TAV aneurysm development. This is consistent with previous findings from Marfan and Loeys-Dietz patients which showed increased nuclear pSMAD2 in medial cells from aneurysmal aortic tissue compared to normal^25^. On the other hand, no significant correlation was observed between pSMAD2 and either TGFß ligand concentration or ascending aortic diameter in BAV or UAV individuals. This could imply SMAD2-independent mechanisms of ascending aortic dilatation in individuals with AV malformations. Furthermore, since hypertension was more prevalent in the TAV group, the relationship between hypertension and canonical TGFß signaling should be further investigated in TAV-related aortic dilatation.

In contrast to pSMAD2, we found that pSMAD3 significantly increased with dilatation in BAV and UAV aortas. As no correlation with TGFß ligand was observed in medial cells, however, SMAD3 activation appears to occur independent of TGFß signaling in BAV and UAV aortas. There is evidence to suggest that activation of SMAD3 may be less dependent upon TGFß signaling compared to activation of SMAD2. For instance, a large portion of intracellular SMAD3, but not SMAD2, resides in the nucleus^26^ and can be recruited to regulatory regions of genes without TGFß signaling^27^. Furthermore, SMAD3 phosphorylation can be initiated by the angiotensin II receptor, AT1, even in the absence of TGFß^28,29^. These observations suggest the possibility that multiple mechanisms independent of canonical TGFß signaling could induce SMAD3 activation. In the present study, a different pattern of SMAD3 activation was observed for TAV aortas, in which pSMAD3 increased with both TGFß ligand concentration and aortic diameter. This implies that TGFß-independent activation of SMAD3 uniquely influences aortic remodeling in BAV/UAV aortas compared to TGFß-driven remodeling in TAV aortas. These findings further emphasize the distinct differences in TGFß signaling between TAV and BAV/UAV aortas, with SMAD3 playing a more deleterious role in BAV/UAV aneurysms.

Despite being younger in age, we found increased elastin breaks/mm^2^ in non-dilated BAV and UAV aortas in comparison to TAV. This suggests the possibility of different mechanisms in BAV and UAV aortas that lead to accelerated aging in the aortic wall compared to TAV aortas. Furthermore, our results are in agreement with a recent study that showed a reduction in aortic elasticity in BAV patients compared to TAV, regardless of whether or not the aorta was dilated^30^. Although the number of elastin breaks/mm^2^ increased with dilatation in all groups, the mechanisms may be different. In TAV aortas, the number of elastin breaks correlated with TGFß ligand concentration, p-SMAD2 and p-SMAD3, suggesting that canonical TGFß signaling could play a role. Furthermore, the majority of individuals with a TAV presented with aortic valve stenosis, which has been associated with up-regulated TGFß ligand expression^31^. This in contrast to the BAV group, where aortic insufficiency was the dominant valve pathology. One possible mechanism could be TGFß1-induced up-regulation of matrix metalloproteinase expression^32^. Interestingly, in BAV and UAV aortas, elastin breaks correlated with pSMAD3, but not pSMAD2 or TGFß ligand concentration. Furthermore, the degree of elastin loss, medial fibrosis and MEMA was increased with higher SMAD3 activation, but not TGFß ligand or SMAD2 activation. This suggests a possible role for SMAD3-specific targets that are involved in extracellular matrix remodeling, such as microRNA 29^33^. Further work is needed to explore the role of SMAD3-dependent extracellular matrix remodeling in BAV and UAV aortas that occurs independent of TGFß. Importantly, the UAV group also had significant aortic stenosis, which could further exacerbate the observed aortic wall degeneration. Whether different mechanisms of aortic wall degeneration exist in individuals with a UAV compared to those with a BAV should be further explored.

In summary, we showed that there are distinct differences in TGFß signaling and SMAD activation in the ascending aorta of individuals with a BAV or UAV compared to those with a TAV. Despite TGFß1 ligand concentration decreasing with dilatation in BAV/UAV aortas, the level of activated SMAD3 increases. SMAD3 activation also increases with dilatation in TAV aortas, but in correlation with increased TGFß1 ligand. Since strategies for therapeutic targeting of the TGFß pathway are being pursued, identifying key factors that preferentially transmit TGFß signals or that independently activate transcription of genes involved in aortic remodeling may provide targets for more effective therapeutic strategies. Our findings reveal that SMAD3 may be a superior therapeutic target against ascending aortic dilatation in general, and maybe even more so in individuals with AV disease.

## Supplementary Information

Supplementary Figures.

## Data Availability

The datasets used and/or analysed during the current study available from the corresponding author on request.
