# Supplementary material for: SMAD3 contributes to ascending aortic dilatation independent of transforming growth factor-beta in bicuspid and unicuspid aortic valve disease
Source: Sci Rep. 2022 Sep 14;12:15476. doi: 10.1038/s41598-022-19335-w (PMC9474869; doi:10.1038/s41598-022-19335-w)
Supplement: Supplementary file 1 — Supplementary Figures. [file 41598_2022_19335_MOESM1_ESM.pdf]

SMAD3 contributes to ascending aortic dilatation independent of transforming growth factor-beta in bicuspid and unicuspid aortic valve disease

Brittany Balint, Jan Federspiel, Catherine Kollmann,
Paul Tepping, Tanja Schwab,
Hans-Joachim Schäfers

Supplemental Data

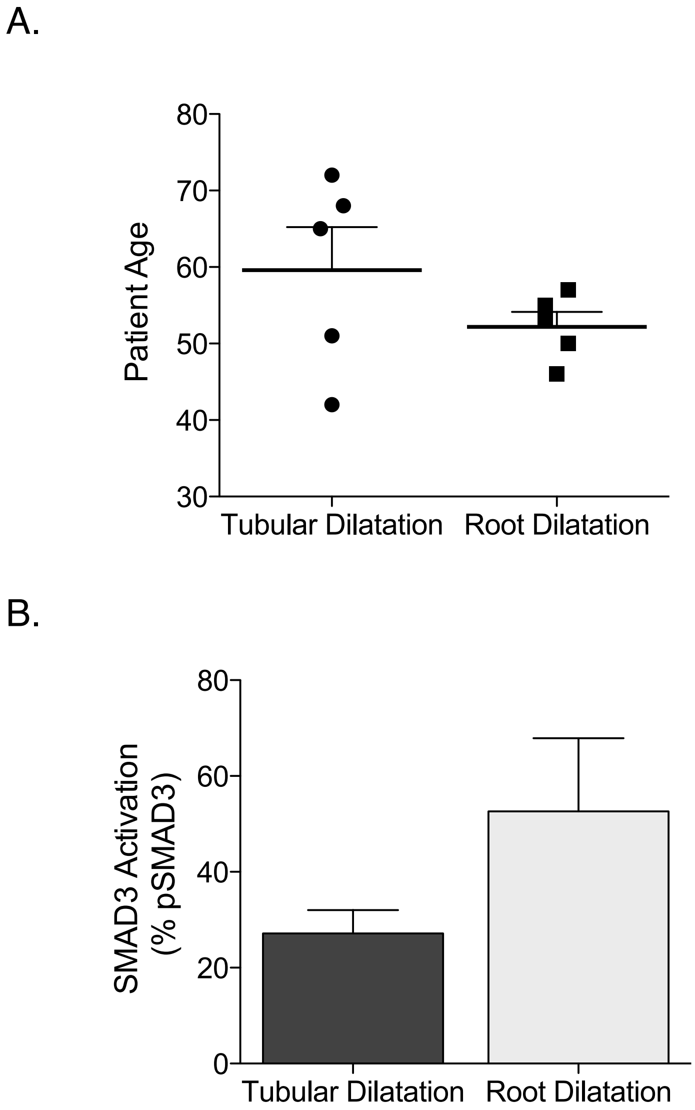

Supplemental Figure 1. Sub-analysis of TAV aneurysms. A. Graph depicting the age of TAV individuals with tubular ascending aortic dilatation (n=5) versus those with an aortic root dilatation (n=5; *P*=0.10). **B.** Graph depicting the level of SMAD3 activation in the ascending aorta of TAV individuals with a tubular dilatation versus those with an aortic root dilatation (*P*=0.08).

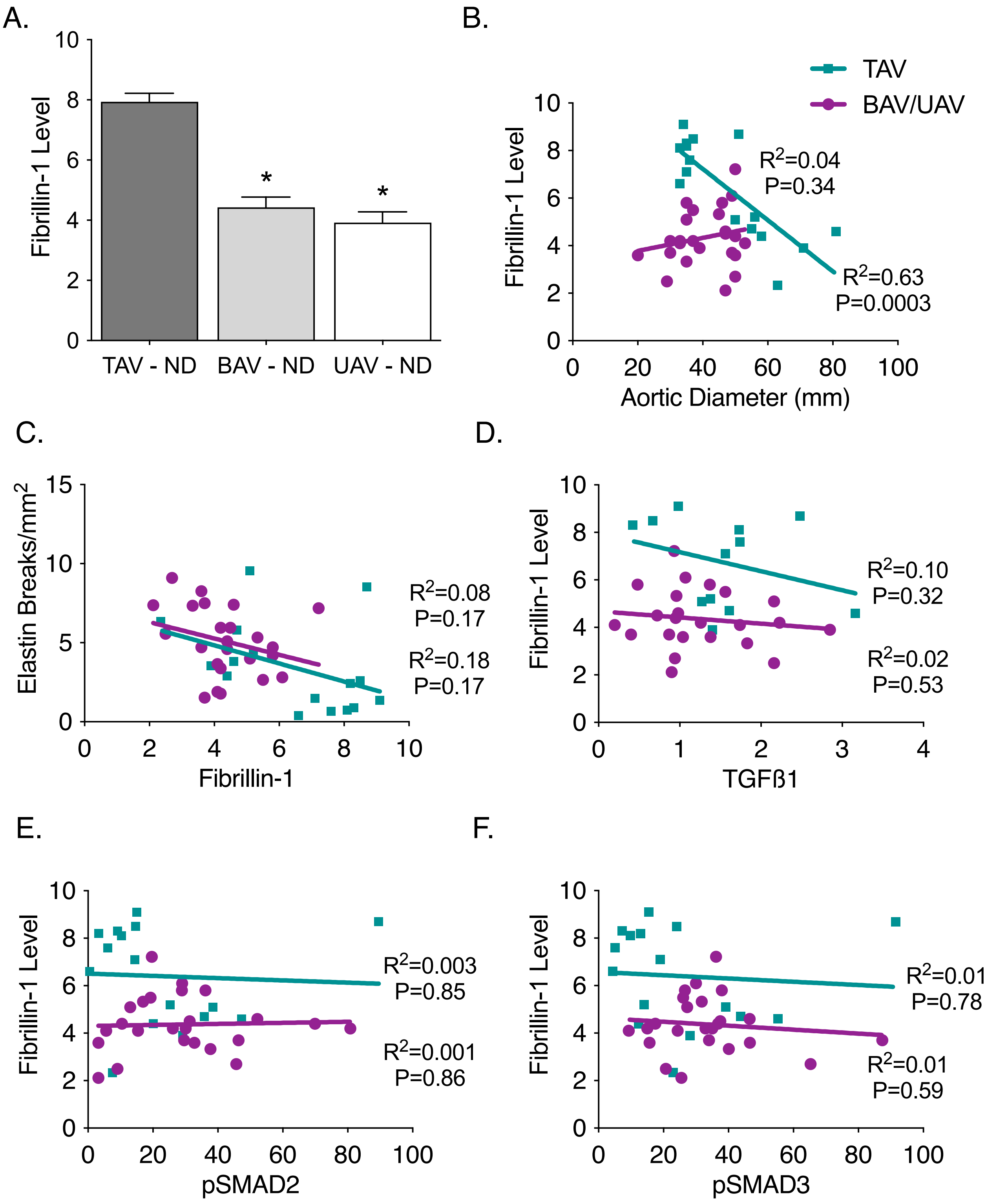

Supplemental Figure 2. Fibrillin-1 concentration. A. Graph depicting the Fibrillin-1 concentration in the aortic media from individuals with non-dilated (ND) aortas and either a tricuspid- (TAV), bicuspid- (BAV) or unicuspid- (UAV) aortic valve. **B-E.** Graphs depicting the relationships between aortic medial Fibrillin-1 levels and maximal aortic diameter **(B)**, elastin breaks (**C**), TGFß1 concentration (**D**), pSMAD2 (**E**), and pSMAD3 (**F**). *=P<0.05.
